# Supplementary material for: Overexpression of Liriodenron WOX5 in Arabidopsis Leads to Ectopic Flower Formation and Altered Root Morphology
Source: Int J Mol Sci. 2023 Jan 4;24(2):906. doi: 10.3390/ijms24020906 (PMC9860802; doi:10.3390/ijms24020906)
Supplement: Supplementary file 1 [file ijms-24-00906-s001.zip › figures s1 and s2.pdf]

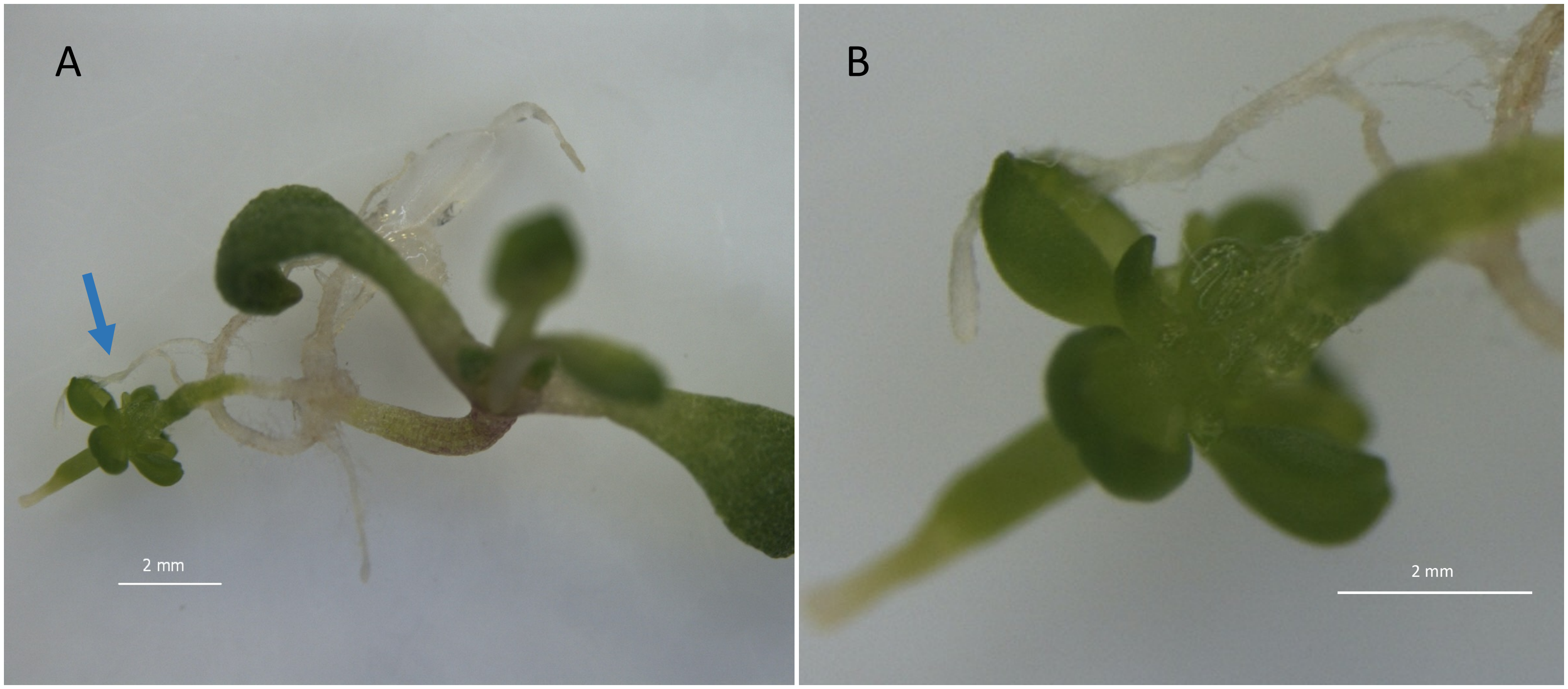

Figure S1. Flower-shaped shoot structure grew on the roots of *p35S:LhWOX5* in *Arabidopsis*.(**A**) The blue arrows show the shoot structure growing on the root. (**B**) Shows enlarged images of A. Scale bars = 2 mm.

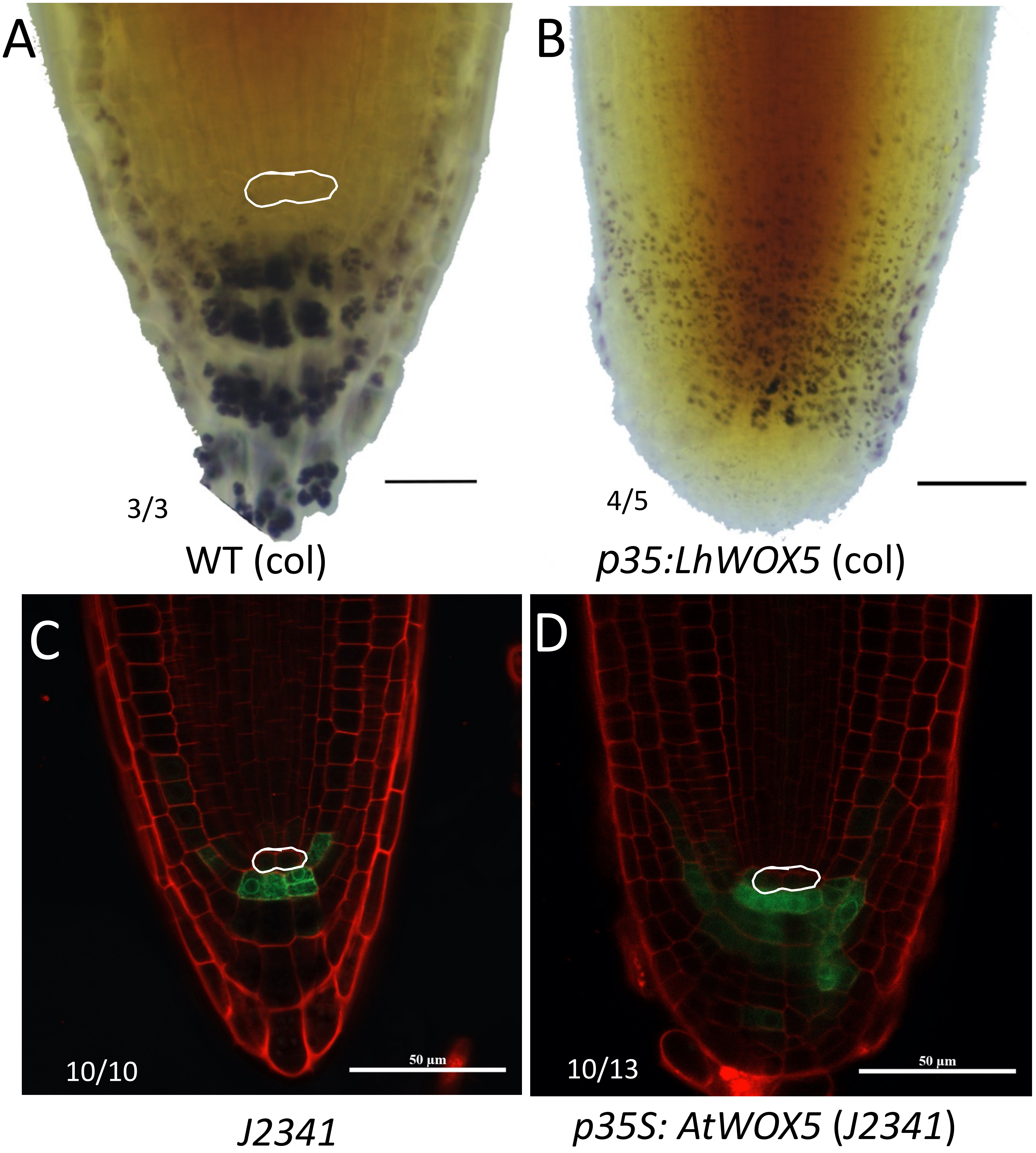

Figure S2. Overexpression of *LhWOX5*/*AtWOX5* results in fewer starch granules and more column stem cells in *Arabidopsis*. (A) WT root meristem stained with lugol solution. (B) *p35: LhWOX5* root meristem with abnormal cells stained with lugol solution. (C) *J2341* (columella stem cell marker) expression pattern. (D) *J2341* expression pattern when *p35S: AtWOX5* was expressed. QC marked in white. Scale bars: A-B, 100 um; C-D, 50 um
